# A Case of Phthisis with a Vomica Communicating with a Gaseous Tumor on the Anterior Aspect of the Chest

**Published:** 1876-10

**Authors:** J. A. Lippincott


### A Case of Phthisis, with a Vomica communicating with a gaseous Tumor on the Anterior Aspect of the Chest, Etc.

By J. A. Lippincott, M. D.

R. C. D., aet. 57, a native of Pennsylvania, and a lumberman by occupation, came under my care May 22, 1876, when the following note in reference to the history of the case was made. The patient, who comes of a healthy family, always enjoyed the best of health until six years ago, when he went through an attack of typhoid fever, which left him with a cough, from which he has never recovered. His illness, however, has not prevented him from working at times in warm weather. Last fall the cough became more severe, and the other symptoms—emaciation, debility, etc.—increased so markedly that he was compelled to take to his bed, where he has remained up to the present time. In August, 1875, the right shoulder began to be painful, stiff, and swollen. The elbow, wrist, and finger-joints were then successively affected. Last February a swelling appeared at the upper part of the right chest, which has been the source of a good deal of pain. This swelling has been steadily enlarging.

I found the patient quite prostrate. He had coated tongue, fetid breath, and anorexia. He was much emaciated, and the capilliary circulation was quite sluggish, as was evidenced by the cyanotic hue of the whole surface. He had a troublesome cough, accompanied by abundant, greenish, sometimes blood-stained, muco-purulent expectoration. The heart-sounds were feeble, but otherwise normal. There were no febrile symptoms, and the urine, with the exception of an excess of urates, contained nothing abnormal. The right shoulder-joint was stiff, and the slightest movement of it caused much pain. The deltoid muscle was flattened so much as to suggest a suspicion of subluxation, but examination showed that the head of the humerus was in its socket, and that the flattening of the muscle was due to atrophy from disuse. A marked degree of false anchylosis was found. The elbow was stiff but movable. The wrist and finger joints—indeed, the whole hand—were very much swollen, and motion was possible in them only to a slight extent.

On inspecting the chest, a uniform swelling was seen on the right side, circular in outline, about five and one-half inches in diameter, projecting about two inches at its centre, and extending from the lower border of the clavicle downwards, and from the right margin of the sternum outwards. It was more decidedly bluish in color than the surrounding parts, tense though elastic to the touch, and tympanitic on percussion. Satisfactory physical exploration of the chest in the region covered by the tumor was impossible, on account of the tenderness of the parts and the debilitated condition of the patient; and for the same reason the chest immediately above the tumor could not be thoroughly examined; but the loud gurgling, cavernous breathing, and tympany elicited over an extended area on very light percussion, demonstrated the existence of a cavity of considerable size and superficial in location. Some impaired percussion resonance, with harsh respiration, prolonged expiration, and a few moist râles, indicated that the upper part of the left lung was also invaded.

Quinine, cream punch, porter, nourishing diet, and a mixture containing dilute muriatic acid and pepsin, were ordered, with an occasional opiate to relieve excessive pain and coughing; and a week later, as the man's condition was considerably improved and as the pain in the tumor seemed to be undiminished, I applied the aspirator and removed the contents, which consisted of inodorous air. After getting rid of the "inflation" and coming down to a "hard" basis, it was seen that the pectoral muscles were atrophied—presumably from the pressure exerted by the contents of the tumor—to such an extent that there now seemed no covering over the ribs other than a cutaneous one. The patient expressed himself as much relieved, and it was possible to make a more careful examination of the chest, which resulted in the conclusion that the vomica extended at least from just below the clavicle on the upper margin of the third rib. After the operation light pressure was applied, but this did not prevent a small degree of extravasation of air into the subcutaneous areolar tissue; this, however, was soon absorbed. Two days after the tapping, slight tumefaction reappeared during a violent fit of coughing, and it has remained since that time, but the swelling has never attained to its original dimensions. At times the communicating fistula seems comparatively pervious, and then the patient can, by pressure, return the contents of the tumor into the pulmonary vomica. I have been able to do this by pressing with the side of my head, and in this manner I have found it possible, by hearing the gurgling noise of the returning air, to fix—approximately at least—the location of the fistula, which is probably at the upper edge of the second rib, about one inch and a half from the margin of the sternum.

The subsequent history of the case is briefly as follows. When the patient had developed sufficient strength and courage, manipulation and friction of the anchylosed joints were instituted and continued, with the result of dissipating the swelling in the wrist and hand and restoring a considerable and most useful degree of motion in all the joints affected. The inflammatory action invaded the left wrist, but subsided quickly after, and possibly on account of, the exhibition of salicylic acid in small quantities dissolved in Huxham's tincture. About the middle of June the patient was able to go out, and also to retain and digest cod-liver oil, and from that time the cough and expectoration have appreciably diminished, and the patient has gained in strength and flesh.

*Remarks.*—The chief point of interest in this case is of course the tumor. Almost all writers speak of the frequency with which pleural adhesions are found when the destruction of lung-tissue is at all extensive, these adhesions being the means by which ulcerations into the pleural cavity, together with the consequent pneumopyothorax, are prevented; but there are comparatively few cases on record in which ulceration beginning on the lungs has extended through the thoracic walls; and no author to whose writings I have access alludes to the possibility of such ulceration resulting in the formation of a circumscribed swelling containing air. Dr. James H. Hutchinson, one of the physicians to the Pennsylvania Hospital, in an article on "The Local Treatment of Pulmonary Cavities by Injection through the Chest-Walls" (*Philadelphia Medical Times* of May 30, 1874), refers to a case—alluded to by "Discipulus," in a series of papers published in Nos. 60, 171 and 180 of the *British Medical Times*—in which a cure is said to have followed the spontaneous evacuation of the contents of a von ica through the walls of the chest. In the same aiticle Dr. Hutchinson quotes a case, seported by M. Bricheteau, in which, as a consequence of the actual cautery to the chest, a pulmonary cavity was opened and empted of its contents·* In the *New York Medical Journal* for January, 1876, appears a brief account of a case of a perforation of the chest-wall in connection with a phthisical cavity. The point of perforation was beneath the clavicle, about two inches from the sternum, and from it, on forcible expiration, air and fluid issued. Before the February number of the *Journal* came out, the patient had died, and at the autopsy a cavity the size of a hen's egg was found, which opened between the first and second ribs. The pleura was adherent around the perforation, but in no other place. Dr. Morris Longstreth has called my attention to a case of "Lungenabscess mit allgemeinem Hautemphyzem," reported by Dr. H. Lenator in *Virchow's Archiv* for 1872, vol. liv. p. 278 :

During the siege of Metz, a Hessian soldier was admitted into hospital for dysentery on the 8th of September. On the 10th, some precussion-dulness was noticed below the inferior angle of the left scapula. The lung-changes went on to softening, which, however, was not detected during life. On the morning of the 17th both sides of the face and neck, the greater part of the trunk in front and behind, and the left arm, were found to be emphysematous. By the 19th, the date of the patient's death, the emphysema had extended over the right arm aud down to the thighs. At the necropsy an abcess, the size of a fist, and surrounded by very firm adhesions, was found at the posterior portion of the lower lobe of the left lung. which communicated with the subcutaneous tissue by at least one small opening situated in the ninth or tenth costal interspace, about two inches from the spinal column.

In this case the ulcerative action, which was acute, rapidly made its way through the deep structures to the subcutaneous space occupied by the loose areolar tissue, whereas, in the others alluded to, the inflammatory process was in all probability sufficiently chronic to permit of the gluing together of the superficial tissues, thus effectually preventing the occurrence of corporeal emphysema. The peculiarity of the case now for the first time reported lies in the fact that the destructive process was so chronic as to allow of the gradual distention, with air, of tissues at least as deep as the fascia lining the greater pectoral muscle.

On first looking at the tumor, and seeing also the swollen joints, the thought flashed across my mind that it was a metastic abcess. This idea rapidly gave way to the suspicion of its being a collec-

*Valleix, Guide du Medecin Praticien, p. 800.

tion of pus communicating with the lung-cavity. A single tap, however, eleciting a distinctly tympanitic sound, revealing the true character of the swelling. That the collection was beneath the deep structures, and not in the subcutaneous areolor tissue, was established by the absence of crepitation, and by the fact that the tumor was circumscribed, being limited at the sternal margin by the origin of the pectoralis major. In fact, before the aspirator was used the pain was most intense along the margin of the sternum, and was doubtless due to the forcible stretching of tendinous fibers of the muscle at its place of origin. The fact that pus did not escape from the vomica into the swelling is to be accounted for by the probable hypothesis that the opening was small and slit-like; and further examination showed very conclusively that it was not at the lower part, but well up on the the anterior wall of the cavity, and that there was a free outlet for the secretions through the bronchial tubes. In view of the following facts, —that the tumor was directly over the vomica (which was extensive); that it was reproduced, after tapping, by violent coughing; that it could be made to disappear by pressure ; and, finally, that on applying the ear to the chest in a certain region, and simultaneously making pressure, the peculiar sound of the air passing into the vomica could be distinctly heard,—it was clear that the pulmonary cavity communicated with the extra-throacic collection of air through an opening in an intercostal space, and constituted, indeed, its *fons et origo*. I man add that Professor Da Costa, who saw the case, lent the weight of his opinion in favor of this conclusion.

As to the local treatment, aspiration was of course indicated as a means of securing temporary relief ; but was no radical treatment to be attempted ? Should I have injected tincture of iodine, or some other irritating fluid, with a view to setting up adhesive inflammation ? I thought not, because, in the first place some of the fluid might find its way into the vomica and interefere with the favorable progress of the case ; and in the second, considering the general debility and the inactivity of the circulation, I feared that suppurative inflammation might be induced which might lead to a communication between the vomica and the external air through the thoracic wall, an event for which I did not feel prepared. There remained what, to my mind, was the safest plan of treatment, namely, by elastic presure ; but the patient preferred to delay the application of the necessary apparatus, and it was not made use of systematically. The tumor, however, has not given him much trouble; if it does, I shall resort to pressure (preceeded, if necessary, by aspiration), by means of raw cotton or soft sponge confined with adhesive strips.

In regard to the administration of salicylic acid, although I have not heard of its being given in cases of pyæmic arthritis, I claim no originality on that score, since the drug has been prescribed of late in so many and so diverse affections, for reasons philosophical an unphilosophical, that it would be strange indeed if it had not long ago been used as a remedy in this form if joint-inflammation.

Since the above was written, the swelling has opened spontaneously. The patient, whom I had not seen for about ten days, sent for me yesterday, and, when I arrived, informed me that for several days he had had a good deal of pain in the tumor, and that "it was hot enough to cook an egg."—a new feature. He also stated that about two hours before my arrival an opening had formed, from which there came a medium-sized cupful of yellowish matter. This had, unfortunately, been thrown out, so that I had not an opportunity to examine it; but his wife said that it was stringy and tenacious. I expressed a small quantity of pus mingled with bubbles of air, and found the former not markedly unhealthy. The opening was over the sternum, near its right edge, on a line with the second intercostal space, some two and a half inches from the point where the aspirating needle had been inserted, which was at the most dependent portion of the swelling. The man was much relieved by the escape of the contents of the tumor, but said he felt faint and slightly nauseated.

What had taken place? Clearly, the intercostal opening, which, for some inexplicable reason, had previously remained small in size, with comparative suddenness—probably during the week—attained sufficient calibre to allow of the passage of the secretions of the vomica. These have produced su ppurative inflammation in the hitherto quiescent tumor, which process has resulted in the usual way. We have, therefore, *a vomica in the lung, communicating with the external air through what may now be regarded as an abcess in the thoracic wall.* I shall probably enlarge the external opening and syringe out the abscess, and thus, indirectly, the vomica, with carbolic or salicylic solutions, and I shall expect to find, in the sputa expectorated, considerable quanties of the material injected.

The further progress of this interesting case I shall at some future time beg the privilege of laying before the readers of the *Times.—Phil. Med. Times.*

